# Retraction: Foxm1 Mediates LIF/Stat3-Dependent Self-Renewal in Mouse Embryonic Stem Cells and Is Essential for the Generation of Induced Pluripotent Stem Cells

**DOI:** 10.1371/journal.pone.0219580

**Published:** 2019-07-05

**Authors:** The PLOS ONE Editors

Following publication of this article [1], concerns were raised regarding Figs 1, 2, and 3:

- Fig 1C Stat 3 panel appears similar to Fig 3B p-Stat3 panel lanes 1–2;
- Fig 2A Stat 3 panel appears similar to Fig 3B p-Stat3 panel lanes 2–3;
- Fig 1C and Fig 2A β-actin panels appear to be duplicated.

The authors explained that β-actin panels in Figs 1C and 2A were derived from the same blot, comprising D3, -LIF, -LIF/+LIF conditions and that the p-Stat3 panel in Fig 3B was published in error and provided a replacement image. The authors conclude that the levels of p-Stat3 are not affected by the Foxm1 knockdown, as reported in the article, even though the signal of Foxm1 shRNA-treated sample appears weaker than that of the untreated sample and is very similar to that of the NC shRNA-treated sample.

In discussing the results with authors, the *PLOS ONE* Editors understand that the western blots in these figures were not normalized to appropriate loading controls. It is also unclear whether replicates were run for the immunoblot analyses.

The authors supplied the uncropped blots for Foxm1, Klf4 and Stat3 figure panels. The blots showed multiple signals for individual antibodies and the bands reported in the published figure correspond to those of approximately the expected molecular weight. The authors explained that they repeated these experiments with similar results, but were unable to provide underlying data to support their claim.

An Academic Editor reviewed the images and noted that owing to the lack of normalization to loading controls, the conclusion around p-Stat3 levels could not be drawn from the data. They also expressed concern at the absence of p-Stat3 signal in the -LIF condition in Fig 1C and Fig 2A. In light of these concerns, that question the validity of the data, the *PLOS ONE* editors retract this article.

GT, LC, and YT did not agree with retraction. TC and LY did not respond.
